# Oxygen administration in patients recovering from cardiac arrest: a narrative review

**DOI:** 10.1186/s40560-020-00477-w

**Published:** 2020-08-12

**Authors:** Ryo Yamamoto, Jo Yoshizawa

**Affiliations:** Department of Emergency and Critical Care Medicine, Keio University School of Medicine, 35 Shinanomachi, Shinjuku, Tokyo, 160-8582 Japan

**Keywords:** Cardiac arrest, Post cardia arrest syndrome, Oxygen, Hyperoxia, Hypoxic brain injury

## Abstract

High oxygen tension in blood and/or tissue affects clinical outcomes in several diseases. Thus, the optimal target PaO_2_ for patients recovering from cardiac arrest (CA) has been extensively examined. Many patients develop hypoxic brain injury after the return of spontaneous circulation (ROSC); this supports the need for oxygen administration in patients after CA. Insufficient oxygen delivery due to decreased blood flow to cerebral tissue during CA results in hypoxic brain injury. By contrast, hyperoxia may increase dissolved oxygen in the blood and, subsequently, generate reactive oxygen species that are harmful to neuronal cells. This secondary brain injury is particularly concerning. Although several clinical studies demonstrated that hyperoxia during post-CA care was associated with poor neurological outcomes, considerable debate is ongoing because of inconsistent results. Potential reasons for the conflicting results include differences in the definition of hyperoxia, the timing of exposure to hyperoxia, and PaO_2_ values used in analyses. Despite the conflicts, exposure to PaO_2_ > 300 mmHg through administration of unnecessary oxygen should be avoided because no obvious benefit has been demonstrated. The feasibility of titrating oxygen administration by targeting SpO_2_ at approximately 94% in patients recovering from CA has been demonstrated in pilot randomized controlled trials (RCTs). Such protocols should be further examined.

## Background

Many patients develop hypoxic brain injury after the return of spontaneous circulation (ROSC), supporting the idea of oxygen administration in patients after cardiac arrest (CA) [1–3]. However, high oxygen tension in blood and/or tissue affects clinical outcomes in multiple diseases [4–6]. Thus, the optimal target PaO_2_ for patients recovering from CA has been extensively examined [7, 8]. Accordingly, the 2015 American Heart Association guidelines for post-CA care recommend decreasing the fraction of inspired oxygen (FiO_2_) when oxyhemoglobin saturation is 100% that can be maintained at 94% or higher [9]. This recommendation indicates that the initiation of oxygen treatment and the amount of oxygen should be deliberately decided. In this review, we described the concept of brain injury following CA, the pathophysiology of hyperoxia, clinical studies of hyperoxia, the practical adjustment of oxygen administration, and ventilatory strategies for resuscitated patients.

## Brain injury after return of spontaneous circulation

Decreased blood blow leads to inadequate oxygen delivery, which cannot maintain the energy demands of the brain after CA, resulting in ischemic insult to brain tissue [2, 10]. Although hypoxia should be managed by high-quality cardiopulmonary resuscitation (CPR) with high-flow oxygen [11, 12], a secondary insult to the brain may also occur after ROSC and is another cause of hypoxic brain injury [13].

This secondary insult is sometimes referred to the “two-hit” model or reperfusion injury by some study groups [10, 13], and pathophysiological mechanisms include endothelial dysfunction, vasogenic cerebral edema, impaired autoregulation of cerebral blood vessels, hyperthermia, and hyperoxia [10, 14–19]. Although extensive research on improvement of clinical outcomes of patients recovering from CA has been conducted, the literature regarding post-cardiac arrest care practices to prevent neuronal cell dysfunction is limited [20–23].

While brain injury after ROSC is mainly due to ischemic insult from decreased cerebral blood flow [1, 2, 10], more than adequate oxygen content in arterial blood also induces neural cell dysfunction [19]. Excessive oxygen produces reactive oxygen species (ROS), such as superoxide, hydrogen peroxide, and hydroxy anion, that can overcome endogenous antioxidants stabilizing cellular function [24]. A systematic review of animal studies demonstrated that neuronal cell dysfunction was induced by normobaric hyperoxia after ROSC [25].

## Pathophysiology of hyperoxia

### Physiology of hyperoxia

Hyperoxia, defined as increased PaO_2_, occurs when the intra-alveolar oxygen partial pressure exceeds the normal breathing condition. After oxygen gas exchange at the alveoli of the lung, most oxygen molecules perfuse into the arterial blood and bind to hemoglobin. Based on the sigmoid-shaped oxygen-hemoglobin dissociation curve, hemoglobin oxygen saturation is determined by oxygen partial pressure in the blood [26, 27]. The remaining oxygen molecules, which are not bound to hemoglobin, are dissolved. According to Henry’s law, there is a linear relationship between oxygen partial pressure and oxygen solubility [28, 29].

The capacity of hemoglobin to bind oxygen molecules is almost saturated (nearly 100%) at normal oxygen partial pressure. Therefore, hyperoxia increases the amount of oxygen dissolved in the blood, resulting in redundant oxygen molecules [24, 28]. When hypothermia is maintained during post-CA care, the solubility of oxygen is increased, and additional redundant oxygen is generated in the blood [29]. Furthermore, hypothermia, as well as alkalosis and hypocarbia, enhances oxygen affinity for hemoglobin (the oxygen-hemoglobin dissociation curve is shifted to the left), and dissolved oxygen accumulates [24, 28–30].

### Redundant oxygen due to hyperoxia

**Fig. 1:**
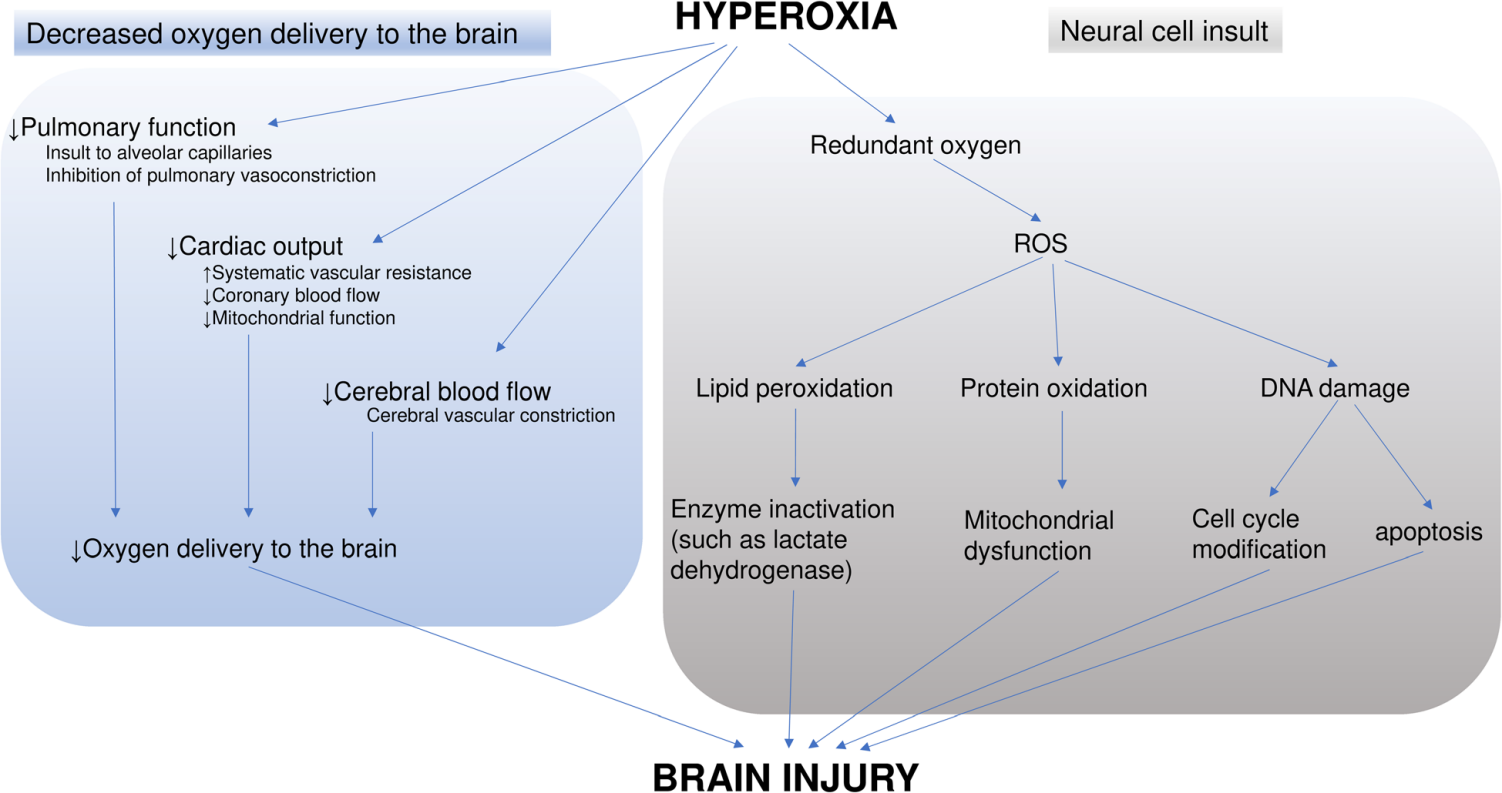
Pathophysiology of hyperoxia. The redundant oxygen resulting from hyperoxia causes overproduction of ROS, which has pathophysiological consequences. ROS induces lipid peroxidation, protein oxidation, DNA damage, which result in neural cell dysfunction. Hyperoxia also causes cerebral vascular constriction, decreased cardiac output, and pulmonary dysfunction, which introduce reductions in oxygen delivery to the brain

The redundant oxygen resulting from hyperoxia causes overproduction of ROS, which has pathophysiological consequences. ROS induces lipid peroxidation and affects cellular membranes that lead to enzyme inactivation and mitochondrial dysfunction [31]. Protein oxidation induced by ROS affects proteolysis [32], and DNA damage due to ROS results in cell cycle modifications and apoptosis [33] (Fig. 1).

Animal studies focused on hyperoxia during post-cardiac care demonstrated that pyruvate dehydrogenase is impaired by ROS, resulting in reduced aerobic metabolism and, subsequently, neuronal cell death [24, 26, 27, 34]. Hyperoxia introduced by 100% FiO_2_ was examined in a CA resuscitation model, and pyruvate dehydrogenase enzyme activity was decreased compared with 21% inspired oxygen [27, 34]. Unfavorable neurological outcomes and neuronal death were also observed in models treated with hyperoxia compared to normoxia in other animal studies [26].

Of note, results from animal studies do not necessarily reflect clinical effects of hyperoxia or pathophysiological derangement due to redundant oxygen in humans; the animal models often lack the simultaneous targeted temperature management, and a wide variety of CA etiology and resuscitation protocols have been utilized in the animal studies [24].

### Hyperoxia and cerebral blood flow

Decreased cerebral blood flow in hyperoxia was also observed in several studies [35, 36]. Although increasing PaO_2_ should theoretically result in greater increases in oxygen delivery to brain tissue, hyperoxia would paradoxically cause reductions in oxygen delivery due to vascular constriction in the brain.

Cardiac output has been also reported to be decreased with hyperoxia, that was induced by increased systemic vascular resistance, decreased coronary blood flow, and impaired mitochondrial function [35, 37, 38]. Redundant ROS with hyperoxia also cause pulmonary toxicity, including insult to alveolar capillaries and inhibition of pulmonary vasoconstriction [39]. These cardiopulmonary dysfunctions would contribute to the brain insult in hyperoxia (Fig. 1).

## Clinical study of hyperoxia

Because of the many discrepant results, it is difficult to identify significant studies that can be clinically adopted for post-CA care. We introduce relevant clinical studies according to the study type and design in chronological order.

### Adverse effects of hyperoxia in retrospective studies

The damaging effects of hyperoxia in animal studies and physiological studies of healthy volunteers are enough to warn physicians to avoid administering oxygen blindly and instigate scientists to explore the optimal target PaO_2_ during post-CA care. Accordingly, considerable number of retrospective studies have reported adverse effects of hyperoxia, such as increased in-hospital mortality, among patients recovering from CA.

In 2010, Emergency Medicine Shock Research Network (EMShockNet) investigators, Kilgannon et al. [7], published a retrospective multicenter cohort study of patients with nontraumatic CA who were resuscitated and admitted to intensive care units (ICUs). In this study, the association of post-resuscitation hyperoxia with increased mortality was examined. A total of 6326 patients were divided into three groups based on PaO_2_ from the first arterial blood gas sample in the ICUs: hyperoxia, ≥ 300 mmHg; normoxia, 300–60 mmHg; and hypoxia, < 60 mmHg or ratio of PaO_2_ to FiO_2_ (P/F ratio) < 300. Patients with hyperoxia had higher in-hospital mortality compared with the normoxia and hypoxia patients (63%, 45%, and 57%, respectively). The association between hyperoxia and unfavorable outcomes remained even after the data were adjusted for potential confounders [7].

In the following year, the same group conducted another retrospective multicenter cohort study on a similar population. However, patients with hypoxia were excluded, and the highest PaO_2_ during the first 24 h in the ICU was examined [40]. A linear trend of increasing in-hospital mortality and decreasing survival depending on PaO_2_ was observed.

Subsequently, several retrospective studies have been published with similar results. In a retrospective analysis by Janz et al. [41], 170 patients recovering from CA were treated with mild therapeutic hypothermia. The highest PaO_2_ in the initial 24 h after resuscitation was lower in the survivors compared to the non-survivors (198 mmHg vs. 254 mmHg), and higher levels of the highest PaO_2_ were independently associated with increased in-hospital mortality (odds ratio [OR]: 1.44; 95% confidence interval: 1.03–2.02). Helmerhorst et al. [42] analyzed 5258 CA patients and compared in-hospital mortality between three different groups defined by PaO_2_ associated with the lowest P/F ratio in the first 24 h. Although hyperoxia (≥ 300 mmHg) was not independently associated with higher mortality compared with normoxia (300–60 mmHg), U-shaped survival curves for PaO_2_ were obtained. Elmer et al. [43] examined 184 patients who suffered from CA at a single center and analyzed the number of hours spent at each PaO_2_ category (severe hyperoxia, > 300 mmHg; moderate or probable hyperoxia, 101–299 mmHg; normoxia, 60–100 mmHg; or hypoxia, <60 mmHg). Severe hyperoxia, but not moderate or probable hyperoxia, was associated with decreased survival after adjustment of known survival predictors. A more recent retrospective study published by Johnson et al. [44] in 2017 also found that hyperoxia (PaO_2_ > 300 mmHg) at 12 h after CA was associated with decreased odds of survival (OR was 0.17 [0.03–0.89]).

### Conflicting results in retrospective studies

While many studies revealed the adverse effects of hyperoxia during post-CA care, several studies have reported conflicting results. Although some of them used the same definition to categorize patients, no significant changes in mortality between patients treated with hyperoxia and those with normoxia were reported.

In a 2011 Study from the Oxygen in Critical Care (SOCC) Group, Bellomo et al. [45], 12,108 patients resuscitated from nontraumatic CA were categorized based on the lowest oxygenation levels during the first 24 h in the ICU (the worst PaO_2_ or PaO_2_ associated with the highest alveolar-arterial gradient). The three groups were the same as those in the EMShockNet study. Although higher in-hospital mortality was observed in patients with hyperoxia compared to that in patients with normoxia (OR: 1.4 [1.3–1.8]), the effect size was decreased after adjusting for illness severity (OR: 1.2 [1.0–1.5]), and the association was not robust in several sensitivity analyses.

In 2013, Ihle et al. [46] analyzed data from 957 patients with out-of-hospital CA (OHCA), which included prehospital data, using the same categories and definitions of oxygenation as the SOCC group. They identified similar in-hospital mortalities between hyperoxia and normoxia (47% vs. 41%), and hyperoxia during the first 24 h in the ICU was not associated with increased mortality (OR: 1.20 [0.51–2.82]). Lee et al. [47] included 213 patients treated with therapeutic hypothermia after CA and categorized patients into four groups based on the distribution of the mean PaO_2_ using quartiles as cut-off values. The mean PaO_2_ was calculated from the entire set of blood gas measurements after ROSC until the end of rewarming. In-hospital mortalities were comparable between patients with the highest mean PaO_2_ (> 156.7 mmHg) and those in the second quartile of PaO_2_ (116.9–134.9 mmHg), although a V-shaped relationship was revealed between the mean PaO_2_ and neurological outcome at hospital discharge. In 2014, Oh et al. [48] investigated hyperoxia within 2 h after ROSC among 792 patients with in-hospital CA and constructed three different groups categorized the same as those in the EMShockNet study. The study indicated that hyperoxia (PaO_2_ ≥ 300 mmHg) was not associated with survival to discharge, compared with normoxia (OR: 1.03 [0.31–3.40]). In a 2017 study published by Auenmueller et al. [49], various values from arterial blood gas samples were collected within 1 h after hospital admission of 170 patients who recovered from OHCA. This study revealed that PaO_2_ was not a survival predictor at 5 days after resuscitation, although insufficient statistical power has been concerned in this study.

Furthermore, Christ et al. [50] analyzed hyperoxia within 1 h after ROSC among 280 OHCA patients. Contrary to other studies, they found that patients with hyperoxia had a statistically higher survival rate than those with normoxia (54% vs. 34%), although the definition of hyperoxia and normoxia was unclear in this study.

### Prospective studies and systematic reviews of hyperoxia

Prospective studies and systematic reviews of hyperoxia in patients resuscitated after CA have also been conducted recently. Notably, two systematic reviews concluded that hyperoxia would be harmful in patients recovering from CA, the meta-analyses included only retrospective studies.

In 2014, Wang et al. [8] conducted a systematic review and meta-analysis of eight retrospective studies, including those we mentioned above, and revealed that hyperoxia, defined as PaO_2_ > 300 mmHg, correlated with increased in-hospital mortality (OR: 1.40 [1.02–1.93]), compared with normoxia.

In the same year, Vaahersalo et al. [51] conducted a multicenter prospective observational study, and neurological outcomes were assessed at 12 months after CA. They recorded arterial blood gas measurements during the first 24 h after ICU admission and categorized PaO_2_ into four groups: low, < 75 mmH; middle, 75–150 mmHg; intermediate, 150–225 mmHg; and high, > 225 mmHg. The proportion of time spent in each oxygen category was calculated for each patient, and no association between the proportion of time spent in the high-PaO_2_ category and better neurological outcome was identified.

Another systematic review was published by Patel et al. [52] in 2018, after the introduction of high-quality CPR, therapeutic hypothermia, and early coronary angiography, which likely led to improvements in clinical outcomes after CA. A meta-analysis of eight retrospective studies demonstrated that hyperoxia, defined as PaO_2_ > 300 mmHg in most studies, was associated with higher mortality (OR: 1.34 [1.08–1.67]). However, the prospective study of 2014 by Vaahersalo et al. was not included in the meta-analysis due to lack of data on mortality.

The most recent prospective observational study was reported by the same study group as the one that conducted the 2018 EMShockNet study, Roberts et al. [53]. A multicenter protocol-directed cohort study of patients who recovered from nontraumatic CA and underwent targeted temperature management was conducted. PaO_2_ was measured 1 and 6 h after ROSC, and hyperoxia was defined as PaO_2_ > 300 mmHg during the initial 6 h. The authors revealed that hyperoxia was independently associated with poor neurological function (relative risk: 1.23 [1.11–1.35]), and the association with poor neurological outcomes began at PaO_2_ ≥ 300 mm Hg.

### Interpretation of differences in clinical studies

Several pathophysiological reasons may account for the conflicting results found in the studies described above (Table 1). First, the definition of hyperoxia was different between the studies. Most of the studies that reported the association of hyperoxia and unfavorable clinical outcomes adopted a definition of PaO_2_ ≥ 300 mmHg or PaO_2_ > 300 mmHg [7, 8, 42–44, 52, 53]. Notably, Elmer et al. reported that severe hyperoxia (> 300 mmHg), but not moderate or probable hyperoxia (101–299 mmHg), was associated with decreased survival [43].

**Table 1:** Differences in clinical studies

	Type of study	Primary outcome	In-hospital mortality		OR or RR for in-hospital mortality	Effect of hyperoxia	PaO2 ≥ (>) 300 mmHg	Hyperoxia ≤ 4–24 h	PaO_2_ value for analysis
			Hyperoxia	Control					
Kilgannon et al. (2010) [7]	Retrospective	In-hospital mortality	63%	45%	1.8 (1.5–2.2)	Unfavorable	Yes	Yes	Highest
Kilgannon et al. (2011) [40]	Retrospective	In-hospital mortality			1.69 (1.56–2.07)	Unfavorable		Yes	Highest
Bellomo et al. (2011) [45]	Retrospective	In-hospital mortality			1.2 (1.0–1.5)	Not significant	Yes	Yes	Lowest
Jannz et al. (2012) [41]	Retrospective	In-hospital mortality	79%	61%	2.53 (1.07–5.96)	Unfavorable	No	Yes	Highest
Ihle et al. (2013) [46]	Retrospective	In-hospital mortality	47%	41%	1.20 (0.51–2.82)	Not significant	Yes	Yes	Lowest
Lee et al. (2014) [47]	Retrospective	In-hospital mortality			0.60 (0.23–1.62)	Not significant	No	No	
Oh et al. (2014) [48]	Retrospective	In-hospital mortality	63%	52%	1.03 (0.31–3.40)	Not significant	Yes	No	Defined timepoint
Wang et al. (2014) [8]	Systematic review	In-hospital mortality			1.40 (1.02–1.93)	Unfavorable	Yes		
Vaahersalo et al. (2014) [51]	Prospective	CPC at 12 months			1.01 (0.998–1.01)	Not significant	No	Yes	
Helmerhorst et al. (2015) [42]	Retrospective	In-hospital mortality	58%	53%	1.10 (0.95–1.27)	Not significant	Yes	Yes	Lowest
Elmer et al. (2015) [43]	Retrospective	In-hospital mortality			1.19 (1.02–1.39)	Unfavorable	Yes	Yes	
Elmer et al. (2015) [43]	Retrospective	In-hospital mortality			0.99 (0.95–1.04)	Not significant	No	Yes	
Johnson et al. (2017) [44]	Retrospective	Neurological function at discharge			5.88 (1.12–33.33)	Unfavorable*	Yes	Yes	Defined timepoint
Auenmueller et al. (2017) [49]	Retrospective	Survival at 5 days			0.85 (0.40–1.83)	Not significant		No	Defined timepoint
Christ et al. (2017) [50]	Retrospective	In-hospital mortality	46%	66%	-	Favorable		No	Defined timepoint
Patel et al. (2018) [52]	Systematic review	In-hospital mortality			1.34 (1.08–1.67)	Unfavorable	Yes**		
Robert et al. (2018) [53]	Prospective	Neurological function at discharge	59%	52%	1.25 (1.01–1.54)	Unfavorable	Yes	Yes	Defined timepoint

*OR* odds ratio, *RR* relative risk, *CPC* cerebral performance category

*Not significant in neurological function at discharge

**One of eight included studies did not use a threshold for defining hyperoxia

Second, the timing of exposure to hyperoxia should be considered. Although hyperoxia within 1–2 h after ROSC was not associated with decreased survival in studies by Oh et al. [48], Auenmueller et al. [49], and Christ et al. [50], exposure to hyperoxia during the first 4–24 h after resuscitation was associated with decreased in-hospital survival rates [40–42, 44, 53]. Of note, Wang et al. [54] demonstrated that hyperoxia at the initial blood gas sample within 24 h after admission was not associated with hospital mortality, whereas later hyperoxia and any hyperoxia were associated with increased hospital mortality (OR: 1.25 [1.11–1.41]) in their retrospective analysis of prospectively collected data.

Finally, the type of PaO_2_ value obtained for analyses was slightly different among the studies. The EMShockNet investigators used PaO_2_ that was obtained at a defined time point or the highest PaO_2_ during the observed period [7, 40, 41], whereas the SOCC group used the lowest PaO_2_ and reported no adverse effect of hyperoxia [45, 46]. Although it is difficult to define hyperoxia in clinical studies, redundant oxygen molecules, rather than higher oxygen partial pressure itself, cause the adverse effects.

## Adjustment of oxygen administration

Although adverse effects of hyperoxia in patients after CA have not been validated, the exposure to PaO_2_ > 300 mmHg due to the administration of unnecessary oxygen should be avoided because no obvious benefit has been reported. In addition, elucidating the optimal amount or a protocol of oxygen administration would be beneficial in providing appropriate post-CA care for physicians.

Eastwood et al. investigated the feasibility of conservative oxygen therapy, in which oxygen was administered with the lowest FiO_2_ to maintain peripheral capillary oxygen saturation (SpO_2_) of 88–92%. In this retrospective analysis of 912 arterial blood gas datasets [55], the median PaO_2_ every 4 h during the initial 24 h after admission was obtained. The authors found that patients treated with conservative oxygen therapy had lower PaO_2_ values and were exposed longer to normoxia (60–120 mmHg), and there was no difference in the proportion of survivors discharged from hospital with good neurological outcome; this suggests that conservative oxygen therapy is physiologically safe for patients following CA [55].

Recently, two pilot studies for RCTs of titrated versus unrestricted oxygen administration after ROSC following CA were reported [56, 57]. The EXACT pilot trial [56] enrolled 61 OHCA patients who achieved and sustained ROSC before hospital arrival. Patients were randomly assigned to either 2–4 L/min oxygen with a target of SpO_2_ ≥ 90% (titrated) or > 10 L/min oxygen (control). Most patients in the study had SpO_2_ ≥ 94% (90% vs. 100%), and all patients had SpO_2_ ≥ 90%, demonstrating the feasibility of oxygen titration in the prehospital environment. The post-ROSC oxygenation (PROXY) study [57] is another pilot study. In this study, 35 patients who had recovered from OHCA were enrolled, and oxygen administration was titrated to a target SpO_2_ of 94–98% and compared to 100% oxygen through the first hour following ROSC in the prehospital environment. Although the authors performed no statistical analyses due to the small sample size, the feasibility of the subsequent RCT was demonstrated.

## Ventilatory strategy for resuscitated patients

Several groups proposed a ventilatory strategy for patients recovering from CA. Newell et al. [58] recommended that the inspired oxygen should be titrated to achieve normal oxygen saturations (94–98%) once oxygenation can be reliably monitored. They also recommended that low tidal volume ventilation (6–8 ml/kg of ideal body weight) should be used with titrated levels of positive end-expiratory pressure to aim for normocapnia [58]. Johnson et al. [59] recommended several goals for ventilator parameters: PaO_2_, 70–100 mmHg; SpO_2_, 92–97%; and tidal volume, 6–8 ml/kg of predicted body weight.

## Conclusions

We reviewed the literature regarding hypoxic brain injury, the pathophysiology of hyperoxia, clinical studies of hyperoxia, and practical adjustment of oxygen administration. Although hypoxic brain injury develops due to insufficient oxygen delivery to cerebral tissue in patients with CA, hyperoxia after ROSC may exacerbate brain injury. Several retrospective studies, a prospective observational study, and two systematic reviews demonstrated that exposure to PaO_2_ > 300 mmHg during post-CA care is associated with unfavorable clinical outcomes, although the results are inconsistent. The feasibility of titrating oxygen administration to target SpO_2_ around 94% in patients after CA has been demonstrated in pilot studies for RCTs. Oxygen administration protocols for patients following CA should be further examined.

## Data Availability

There was no dataset used in this study.

## Abbreviations

CA: Cardiac arrest
ROSC: Return of spontaneous circulation
PaO_2_: Arterial oxygen partial pressure
CPR: Cardiopulmonary resuscitation
ROS: Reactive oxygen species
FiO_2_: Fraction of inspired oxygen
EMShockNet: Emergency Medicine Shock Research Network
ICU: Intensive care unit
P/F ratio: Ratio of PaO_2_ to FiO_2_
OR: Odds ratio
SOCC: Study of oxygen in critical care
OHCA: Out-of-hospital cardiac arrest
SpO_2_: Peripheral capillary oxygen saturation
PROXY: Post-ROSC oxygenation
